# Adapting the preterm birth phenotyping framework to a low-resource, rural setting and applying it to births from Migori County in western Kenya

**DOI:** 10.1186/s12884-023-06012-7

**Published:** 2023-10-16

**Authors:** Lara Miller, Christina N. Schmidt, Phillip Wanduru, Anthony Wanyoro, Nicole Santos, Elizabeth Butrick, Felicia Lester, Phelgona Otieno, Dilys Walker

**Affiliations:** 1 University of California San Francisco, Institute for Global Health Sciences, 550 16Th St, San Francisco, CA 94158 USA; 2 University of California San Francisco, School of Medicine, 533 Parnassus Ave, San Francisco, CA 94143 USA; 3 School of Public Health, Makerere University, New Mulago Gate Rd, Kampala, Uganda; 4 Department of Obstetrics and Gynaecology, Kenyatta University, Main Campus, Kenya Drive, Nairobi, Kenya; 5 Department of Obstetrics, University of California San Francisco, Gynaecology & Reproductive Sciences, 1825 Fourth St Third Floor, San Francisco, CA 94158 USA; 6 Kenya Medical Research Institute, 00200 Off Raila Odinga Way, Nairobi, Kenya

**Keywords:** Premature birth, Phenotype, Maternal infection, Perinatal mortality

## Abstract

**Background:**

Preterm birth is the leading cause of neonatal and under-five mortality worldwide. It is a complex syndrome characterized by numerous etiologic pathways shaped by both maternal and fetal factors. To better understand preterm birth trends, the Global Alliance to Prevent Prematurity and Stillbirth published the preterm birth phenotyping framework in 2012 followed by an application of the model to a global dataset in 2015 by Barros, et al. Our objective was to adapt the preterm birth phenotyping framework to retrospective data from a low-resource, rural setting and then apply the adapted framework to a cohort of women from Migori, Kenya.

**Methods:**

This was a single centre, observational, retrospective chart review of eligible births from November 2015 – March 2017 at Migori County Referral Hospital. Adaptations were made to accommodate limited diagnostic capabilities and data accuracy concerns. Prevalence of the phenotyping conditions were calculated as well as odds of adverse outcomes.

**Results:**

Three hundred eighty-seven eligible births were included in our study. The largest phenotype group was none (no phenotype could be identified; 41.1%), followed by extrauterine infection (25.1%), and antepartum stillbirth (16.7%). Extrauterine infections included HIV (75.3%), urinary tract infections (24.7%), malaria (4.1%), syphilis (3.1%), and general infection (3.1%). Severe maternal condition was ranked fourth (15.6%) and included anaemia (69.5%), chronic respiratory distress (22.0%), chronic hypertension prior to pregnancy (5.1%), diabetes (3.4%), epilepsy (3.4%), and sickle cell disease (1.7%). Fetal anaemia cases were the most likely to transfer to the newborn unit (OR 5.1, 95% CI 0.8, 30.9) and fetal anomaly cases were the most likely to result in a pre-discharge mortality (OR 3.9, 95% CI 0.8, 19.2).

**Conclusions:**

Using routine data sources allowed for a retrospective analysis of an existing dataset, requiring less time and fewer resources than a prospective study and demonstrating a feasible approach to preterm phenotyping for use in low-resource settings to inform local prevention strategies.

## Abstract (plain language summary)

Preterm birth is a complex syndrome, yet it is the leading cause of death in children worldwide. To help unravel the clinical complexities, preterm birth phenotyping is a framework that considers multiple diagnoses in the mother, helping to evaluate trends in causes of preterm birth in a given region. In our study, we adapted this international phenotyping framework to accommodate a rural, low-resource setting where obstetrical and neonatal technologies were limited, but preterm birth rates were high. We evaluated data from the patient records of a large hospital in Migori, Kenya, in the southwestern region of the country. By lowering the threshold of diagnostic criteria, we were able to apply this framework to our dataset and see that maternal infection and maternal chronic illness appear to be a significant driving forces of preterm birth. Given high rates of HIV and malaria in the region, this is not a surprising finding, but one that can inform antenatal care practices, mainly the need to test and treat for common infections (HIV, malaria, as well as urinary and reproductive tract infections), and to increase the frequency of antenatal care interactions per the World Health Organization recommendations.

## Background

Resulting in the death of one million children per year, preterm birthis the leading cause of both neonatal and under-five mortality worldwide [1]. Preterm birth, defined as birth before 37 weeks of gestation, is a complex syndrome characterized by numerous etiologic pathways inclusive of both maternal and fetal contributing factors. The cause of a preterm birth may be the result of one independent factor or the interplay of multiple factors [2]. Biologic indicators causally linked to preterm birth include extrauterine infection, chorioamnionitis, maternal chronic illness (such as diabetes, hypertension previous to pregnancy, epilepsy, etc.), multiparous pregnancy, intrauterine growth restriction (IUGR), fetal sepsis, fetal anaemia, fetal distress, fetal anomaly, placental abnormalities, antepartum stillbirth, and pre-eclampsia/eclampsia [3].

In 2012, the Global Alliance to Prevent Prematurity and Stillbirth (GAPPS) released a classification system for preterm birth based on “clinical phenotypes” to consider multiple causal factors that may lead to a preterm delivery. Their preterm birth phenotypes were defined as “one or more significant conditions related to the mother, the foetus, and/or the placenta that are present before initiation of parturition” [4]. The GAPPS framework distinguishes between a risk factor and a clinical phenotype by stating that the relationship between preterm delivery and a clinical phenotype must be causal, not simply correlative, meaning factors such as age, socioeconomic status, smoking, and high stress levels are not considered phenotypes. This system allows for the consideration of all biologic factors that may contribute to a preterm birth and recognizes the interplay of multiple conditions present in one pregnancy. It is a wider lens through which to analyse preterm birth trends in a given area, and ultimately inform future intervention priorities [5].

In 2015, Barros et al. applied the GAPPS system to preterm births from the INTERGROWTH-21^st^dataset and through a 2-step cluster analysis identified twelve preterm birth phenotypic clusters and their prevalence across eight countries [3]. The study populations, however, were women in urban areas, with adequate antenatal care and early ultrasound data, reflecting a population with generally lower preterm birth rates. Our analysis sought to adapt and apply the Barros et. al phenotyping framework to a cohort of women from a rural, low-resource facility in Migori, Kenya using only routine data sources. After adapting the framework, we evaluated the most common preterm birth phenotypes and determined which were most strongly associated with newborn morbidity and mortality.

## Methods

### Study design and setting

This study was nested in the University of California, San Francisco (UCSF) East Africa Preterm Birth Initiative’s (PTBi-EA) intrapartum quality improvement cluster-randomized control trial [6]. It was a single centre, observational, retrospective chart review of all eligible preterm births from November 2015 – March 2017 at Migori County Referral Hospital (MCRH). The institutional review boards of UCSF (Study ID# 16–19,162) and the Kenya Medical Research Institute (KEMRI) (Study ID# 0034/321) reviewed and approved this study and all methods were carried out in accordance with relevant guidelines and regulations.

Migori County is in the southwestern region of Kenya, on the border with Tanzania. MCRH is a level IV government referral hospital with approximately 4,000 births per year and receives most of the region’s complicated maternal and neonatal cases.

### Adapting the Barros framework

The Barros et al. definitions were adapted to allow for missed diagnoses, limited data availability, and variations in the completeness and accuracy of data in a low-resource setting. From the available data, we were able to adapt nine of the twelve phenotyping conditions used in Barros et al. HELLP syndrome, IUGR suspicion, and early pregnancy bleeding were unable to be identified in this setting due to limited laboratory data, low and late antenatal care (ANC) engagement, inconsistent recording of ANC data, and variable gestational age (GA) accuracy. We added fetal anaemia as a phenotype, due to the high prevalence of this condition in our study population which was not included in Barros et al. as a phenotype but was listed as a fetal condition with a causal relationship to preterm birth. Finally, sickle cell anemia and other documented severe anemia not related to the presence of malaria infection were added to the phenotype “severe maternal condition” due to the high prevalence of these conditions in our sample population. Table 1 explains the adapted definitions in detail.

**Table 1:** Phenotypic conditions with diagnostic criteria adapted to a low-income setting

	**Barros Definition**	**Adapted Definition**
**Maternal Conditions**		
Chorioamnionitis	Cases where antibiotic treatment was specifically indicated for preterm premature rupture of membranes (PPROM). Suspected chorioamnionitis cases with intact membranes were not possible to identify in this data set	Stated as a diagnosis **OR** PPROM and presence of at least one of the following **OR** no PPROM and presence of at least two of the following without documented extrauterine infection: one measured maternal temperature > 38 °C, maternal tachycardia (≥ 100 beats per minute), leukocytosis (leukocytes ≥ + in urinalysis), fetal tachycardia (> 160 beats per minute), maternal bradycardia (< 60 bpm), uterine tenderness, foul-smelling and/or green amniotic fluid and/or cervical discharge
Extrauterine infection	Presence of at least 1 of the following: malaria, pyelonephritis. sexually transmitted diseases (including syphilis and HIV/AIDS), and other clinically documented infections that required use of antibiotics or other treatments during pregnancy, except when antibiotics were used for PPROM	*Human immunodeficiency virus (HIV):* Positive HIV test (rapid test, enzyme-link immunosorbent assay or viral culture) **OR** documented known HIV positive **OR** maternal ARV use **OR** neonatal nevirapine administration. *General Infection:* Unspecified fever > 38 °C. *Malaria:* Stated diagnosis **OR** merozoite surface protein (MSP) presence in blood panel. *Syphilis:* Stated diagnosis **OR** one reactive/positive Venereal Disease Research Laboratory (VDRL) test. *Urinary Tract Infection:* Stated diagnosis **OR** one of the following on urinalysis: leukocytes + + + / + + , and/or pus cells + + + / + + , and/or epithelial cells + + + / + +
Pre-eclampsia	Defined as elevated blood pressure (≥ 140/90 mm Hg), 30 mm Hg increase of systolic pressure, or 15 mm Hg increase of diastolic pressure in relation to basal measurements observed at least twice, the interval of the measurements being > 4 h but < 168 h and proteinuria > 2 + by dipstick	Stated diagnosis **OR** MgSO4 administration **OR** at least one documented blood pressure ≥ 140/90 mmHg and one or more of the following **OR** no documented blood pressures and two or more of the following: + protein or greater in a urinalysis, blurry vision, severe headache, lower extremities edema, oligohydramnios without PPROM
Severe maternal condition (clinically active during the index pregnancy)	Cases with a relevant clinical condition documented in the medical records in which birth was caregiver initiated because of the severity or complications related to these conditions. This excludes cases in which there was also an obstetric reason for induction/cesarean delivery. Clinical conditions associated with caregiver-initiated preterm birth included diabetes mellitus, thyroid disease, other endocrine diseases, cardiac disease, hypertension previous to pregnancy, chronic respiratory disease (including chronic asthma), renal disease, cancer, lupus erythematosus, any coagulopathy (including falciparum anemia), tuberculosis, severe intestinal malabsorption (including Crohn and celiac diseases), maternal congenital abnormality or genetic disease (e.g., cystic fibrosis or cardiac congenital defects), epilepsy, or any other clinical condition that required surgery or referral to specialized care	Presence of one or more of the following conditions: maternal anemia (Hg < 11 g/dL), diabetes mellitus, thyroid disease, other endocrine diseases, cardiac disease, hypertension previous to pregnancy, chronic respiratory disease (including chronic asthma), renal disease, cancer, lupus erythematosus, any coagulopathy (including falciparum anemia), tuberculosis, severe intestinal malabsorption (including Crohn’s and celiac diseases), maternal congenital abnormality or genetic disease (e.g. cystic fibrosis or cardiac congenital defects), epilepsy, or any other clinical condition that required surgery or referral to specialized care. Specific additions of sickle cell anemia and other documented severe anemia not related to the presence of malaria infection
HELLP Syndrome	HELLP (hemolysis, elevated liver enzymes and low platlets) or any other coagulation abnormalities reported from a pregnant woman with pre-eclampsia or eclampsia	Unable to be diagnosed in this setting
**Fetal Conditions**		
Antepartum stillbirth	All fetal deaths occurring before the clinically reported start of labor	Documented macerated stillbirth **OR** diagnosis of intrauterine fetal death
Fetal anemia	For example, due to fetal hemolytic disease; Rhesus negative	Stated diagnosis **OR** documented maternal rhesus negative status
Fetal anomaly	Severe anomalies diagnosed through pregnancy ultrasonography or on neonatal examination	Severe anomalies documented post-partum
Fetal distress	Diagnosis based on: (1) abnormal antepartum nonstress test reported in the medical record as indication for induction of labor or elective cesarean delivery or (2) severe intrapartum electronic fetal monitoring pattern equivalent to category 3 of NICHD as indication for intrapartum cesarean delivery	Stated diagnosis **OR** presence of meconium **OR** fetal tachycardia (≥ 160 beats per minute) or fetal bradycardia (< 110 beats per minute)
Intrauterine growth restriction suspicion	Suspicion of impaired fetal growth during pregnancy based on ultrasonography examinations or physical examination and specifically stated in the medical record	Unable to be diagnosed in this setting due to questionable quality of gestational age and limited prenatal ultrasound completion
Multiple births	≥ 2 Fetuses in the same pregnancy	≥ 2 Fetuses in the same pregnancy
**Fetal conditions**		
Early pregnancy bleeding	Vaginal bleeding < 15^+0^ weeks’ gestation	Unable to be diagnosed in this setting due to limited antenatal care data and few women seeking care in the first trimester
Mid-to-late pregnancy bleeding	Vaginal bleeding ≥ 15^+0^ weeks’ gestation without the diagnosis of pre-eclampsia, eclampsia, or HELLP syndrome	Vaginal bleeding ≥ 15 weeks’ gestation either as a documented diagnosis or as any bleeding prior to rupture of membranes **OR** specific diagnosis of placenta previa or placental abruption
Third trimester bleeding and pre-eclampsia	Vaginal bleeding occurring > 27^+0^ weeks’ gestation in women diagnosed as having severe pre-eclampsia, eclampsia, or HELLP syndrome	Pre-eclampsia or eclampsia **AND** antepartum bleeding, placenta previa or placental abruption
**Outcomes Definitions**		
Pre-discharge mortality	Babies born alive but with a documented mortality before the time of discharge **OR** 5- or 10- minutes APGAR scores equal to 0 (resuscitation is typically unsuccessful in this setting)	

### Data collection

Due to a lack of early pregnancy ultrasounds, inconsistent recording of GA data (i.e., 32 weeks in one area of the chart and 34 weeks in another), and unlikely GA and birth weight combinations (i.e. 4000 g and 28 weeks), GA accuracy was determined to be limited in this setting. As such, eligibility criteria were based on a combination of listed GA and birthweight, including: 1) all babies with a birth weight less than 2500 g, and 2) and babies with a birth weight between 2500 to 3000 g only if the GA recorded in the MR was < 37 weeks. As babies greater than 3000 g were most likely term and those less than 2500 g were most likely preterm, recorded GA was used only for babies who fell within the more uncertain 2500-3000 g range. These definitions are consistent with the eligibility criteria defined by the PTBi-EA parent study and are explained in greater detail elsewhere [7, 8].

Eligible births were identified in the MCRH maternity register (MR), a Ministry of Health logbook where maternity demographic and outcomes data are hand-recorded for each birth by the attending healthcare provider. The inpatient number associated with each eligible MR record was recorded, and the corresponding Maternity Unit inpatient record (IPR) was retrieved from the on-site health records office. If an IPR could not be found, the birth was excluded from the analysis.

Data were extracted from the MR and IPR by PTBi-EA research staff and entered into an Open Data Kit (ODK) tool. The variables extracted were predetermined in consultation with obstetricians and paediatricians from UCSF and KEMRI and modelled on the Barros et al. phenotypic cluster diagnostic criteria. If data between the MR and IPR contradicted one another, preference was given to the IPR.

### Data analysis

The clinical data for each birth were reviewed and classified into one or more of the phenotypes by two independent researchers (LM and CS). For each phenotype, births were categorized as having either a single condition or multiple conditions. Extrauterine infection and severe maternal condition were further disaggregated to report the breakdown of infections and conditions. The frequency of each phenotype within the study population was calculated, as well as the frequency of corresponding conditions within each phenotypic subgroup.

To evaluate outcomes associated with each phenotype, the mean birthweights, GAs, parity, maternal age, and antenatal care (ANC) visits were calculated, as well as the proportion and odds ratios of babies who were transferred to the special care newborn unit (NBU), or died before hospital discharge (pre-discharge mortality, PDM). All data were analysed in Microsoft Excel (version 16.16.3) and RStudio (Version 1.0.136).

### Ethical considerations

All data were extracted from routine data sources collected from patients by healthcare providers. No personally identifiable data were collected, and all data were stored on encrypted computers and servers. Permission to access the data was sought and granted by the leadership of MCRH.

## Results

**Fig. 1:**
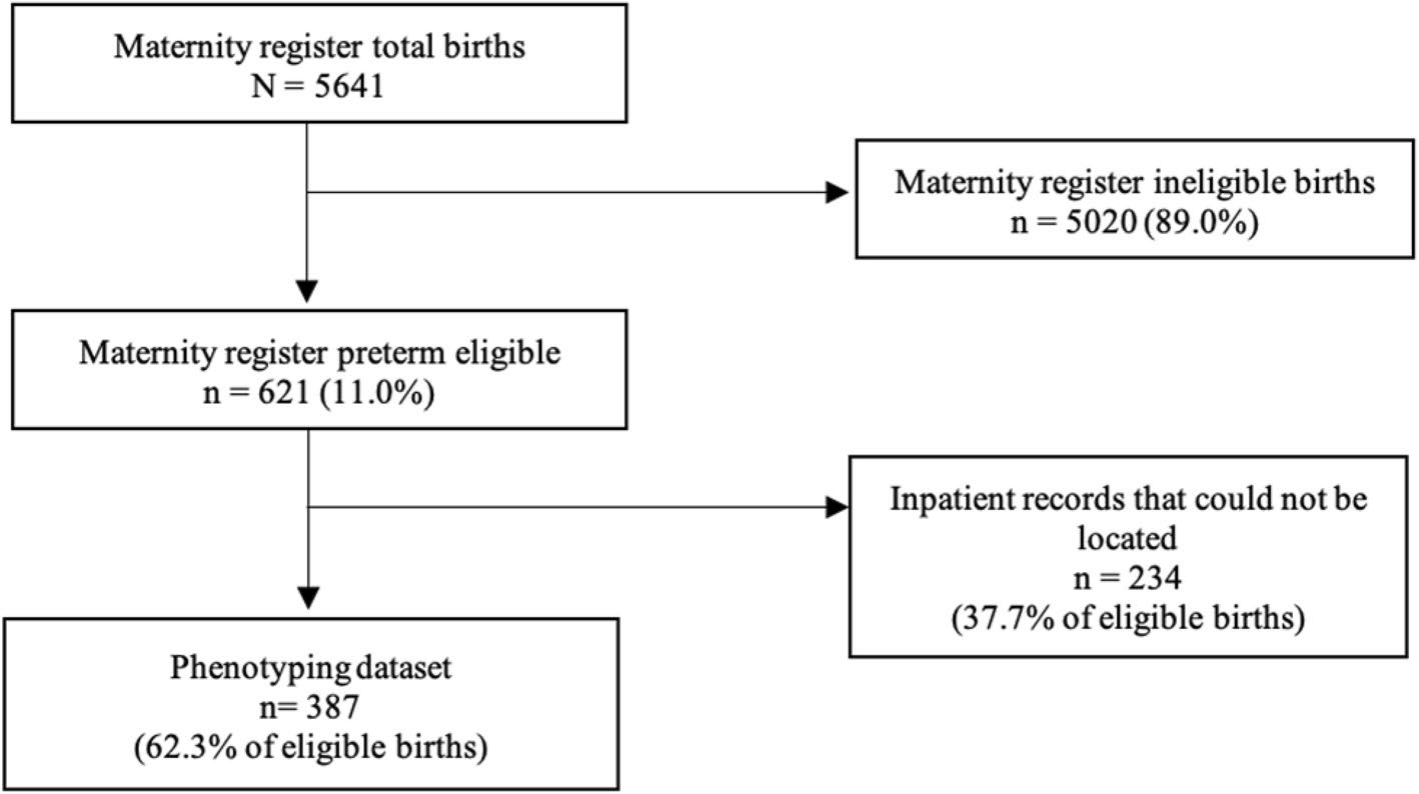
Preterm birth phenotyping eligibility flow chart at Migori County Referral Hospital

From November 2015 to May 2017 there were 5,641 births at MCRH of which 621 (11.0%) met the eligibility criteria. Of eligible births, 234 (37.7%) IPRs could not be traced, resulting in a dataset of 387 (Fig. 1).

Of eligible women with documented socio-demographic information, the majority were 20 – 26 years old, married, without formal employment, and with a primary school level of education. Women received an average of 3 ANC visits and had an average of 1.4 deliveries prior to the index pregnancy (Table 2).

**Table 2:** Demographic information of maternity unit patients at Migori County Referral Hospital

Total N	387
**Demographics (%)**	
**Maternal Age**	
13 – 19	28.2
20 – 26	34.4
27 – 33	16.0
34 – 43	6.5
Not listed	15.0
**Marital status**	
Married	61.2
Unmarried	16.5
Not listed	22.2
**Education**	
Primary	15.2
Secondary	6.7
University	6.5
Not listed	71.6
**Occupation**	
Student	7.2
Subsistence farming	8.5
Employed	16.8
Not formally employed	23.0
Not listed	44.4
**Birth Data (means and ranges)**	
Parity^a^	1 [0 – 9]
Gravidity^a^	3 [1–10]
Antenatal care attendance	3 [1–8]
Birth weight (grams)	2116 [400 – 2900]
Gestational age (weeks)	33 [15 – 43]

^a^Parity is reported prior to the index pregnancy. Gravidity includes the index pregnancy

Table 3 summarizes the preterm birth phenotypes of the cohort. The largest group was none (no phenotype was identified) at 41.1%, followed by extrauterine infection (25.1%), and antepartum stillbirth (16.7%). Extrauterine infection represented cases of HIV (75.3%), urinary tract infections (UTI) (24.7%), malaria (4.1%), syphilis (3.1%), and general infection (3.1%). Severe maternal condition was ranked fourth (15.6%) and included diagnoses of anaemia (69.5%), chronic respiratory distress (22.0%), chronic hypertension prior to pregnancy (5.1%), diabetes (3.4%), epilepsy (3.4%), and sickle cell disease (1.7%). Corresponding conditions showed trends between various phenotyping groups. Of cases with fetal anaemia, 80.0% also had a severe maternal condition. Of cases with chorioamnionitis, 60.0% resulted in an antepartum stillbirth. Of severe maternal conditions cases, 40.0% also had an extrauterine infection.

**Table 3:** Phenotypic distribution of eligible babies at Migori County Referral Hospital

**Phenotype**^a^** ( *N* = 387)**	**n (%)**	**Single Condition**	**Multiple Conditions**	**Corresponding Conditions**^b^
**1. None**	**158 (41.1%)**	**NA**	**NA**	NA
**2. Extrauterine infection**	**97 (25.1%)**	**37 (38.1%)**	**60 (61.9%)**	Severe maternal condition (25.8%), antepartum stillbirth (19.6%), preeclampsia (9.3%), multiple gestation (9.3%), mid-to-late pregnancy bleeding (8.2%), fetal anomaly (6.21%), fetal distress (5.2%), chorioamnionitis (6.2%), fetal anomaly 6.2%)
HIV^a^	73 (76.8%)	60 (82.2%)	13 (17.8%)	Urinary tract infection (8.2%), syphilis (2.7%), malaria (1.4%), general infection (1.4%)
Urinary tract infection	24 (25.3%)	18 (75.0%)	6 (25.0%)	HIV (33.3%), malaria (8.3%), general infection (4.2%)
Malaria	4 (4.2%)	2 (50.0%)	2 (50.0%)	Urinary tract infection (50.0%), HIV (25.0%)
Syphilis	3 (3.2%)	1 (33.3%)	2 (66.7%)	HIV (66.7%)
General infection	3 (3.2%)	1 (33.3%)	2 (66.7%)	HIV (33.3%), urinary tract infection (33.3%)
**3. Antepartum stillbirth**	**63 (16.7%)**	**26 (41.3%)**	**37 (58.7%)**	Extrauterine infection (30.2%), severe maternal condition (23.8%), preeclampsia (14.3%), mid-to-late pregnancy bleeding (12.7%), chorioamnionitis (12.7%), multiple gestation (4.8%), fetal anomaly (3.2%), fetal anemia (1.6%)
**4. Severe maternal condition**	**59 (15.6%)**	**16 (27.1%)**	**43 (72.9%)**	Extrauterine infection (42.4%), antepartum stillbirth (25.4%), mid-to-late pregnancy bleeding (16.9%), preeclampsia (10.2%), fetal distress (6.8%), multiple gestation (6.8%), chorioamnionitis (8.5%), fetal anomaly (1.7%)
Anemia^a^	41 (70.7%)	37 (90.2%)	4 (0.1%)	Chronic respiratory distress (4.9%), hypertension prior to pregnancy (2.4%), epilepsy (2.4%)
Chronic respiratory distress	13 (22.4%)	11 (84.6%)	2 (15.4%)	Anemia (15.4%)
Hypertension prior to pregnancy	3 (5.2%)	2 (66.7%)	1 (33.3%)	Anemia (33.3%)
Diabetes	2 (3.4%)	2 (100.0%)	-	-
Epilepsy	2 (3.4%)	1 (50.0%)	1 (50.0%)	Anemia (50.0%)
Sickle cell	1 (1.7%)	1 (100.0%)	-	-
**5. Multiple births**	**47 (12.4%)**	**30 (63.8%)**	**17 (36.2%)**	Extrauterine infection (19.1%), preeclampsia (8.5%), severe maternal condition (8.5%), antepartum stillbirth (6.4%), mid-to-late pregnancy bleeding (2.1%)
**6. Preeclampsia**	**31 (8.2%)**	**8 (25.8%)**	**23 (74.2%)**	Antepartum stillbirth (29.0%), extrauterine infection (29.0%), severe maternal condition (19.4%), multiple gestation (12.9%), chorioamnionitis (16.1%), fetal distress (9.7%), mid-to-late pregnancy bleeding (3.2%), fetal anomaly (3.2%)
**7. Mid-to-late pregnancy bleeding**	**29 (7.7%)**	**10 (34.5%)**	**19 (65.5%)**	Severe maternal condition (34.5%), antepartum stillbirth (27.6%), extrauterine infection (27.6%), chorioamnionitis (6.9%), fetal anomaly (3.4%), fetal distress (3.4%), multiple gestation (3.8%), preeclampsia (3.4%)
**8. Fetal distress**	**14 (3.7%)**	**5 (35.7%)**	**9 (64.3%)**	Extrauterine infection (35.7%), severe maternal condition (28.6%), preeclampsia (21.4%), fetal anomaly (7.1%), chorioamnionitis (7.1%), mid-to-late pregnancy bleeding (7.1%)
**9. Chorioamnionitis**	**13 (3.4%)**	**1 (7.7%)**	**12 (92.3%)**	Antepartum stillbirth (61.5%), extrauterine infection (46.2%), severe maternal condition (38.5%), preeclampsia (38.5%), mid-to-late pregnancy bleeding (15.4%), fetal distress (7.7%), fetal anomaly (7.7%), fetal anemia (7.7%)
**10. Fetal anomaly**	**10 (2.6%)**	**2 (20.0%)**	**8 (80.0%)**	Extrauterine infection (60.0%), antepartum stillbirth (20.0%), fetal distress (10.0%), preeclampsia (10.0%), mid-to-late pregnancy bleeding (10.0%), severe maternal condition (10.0%), chorioamnionitis (10.0%)
**11. Fetal anemia**	**2 (0.5%)**	**1 (50.0%)**	**1 (50.0%)**	Chorioamnionitis (50.0%), antepartum stillbirth (50.0%)

^a^Number of phenotypes: one (37.0%), two (20.7%), three (7.5%), four (3.1%), and five (1.8%)

^b^Conditions not mutually exclusive

**Fig. 2:**
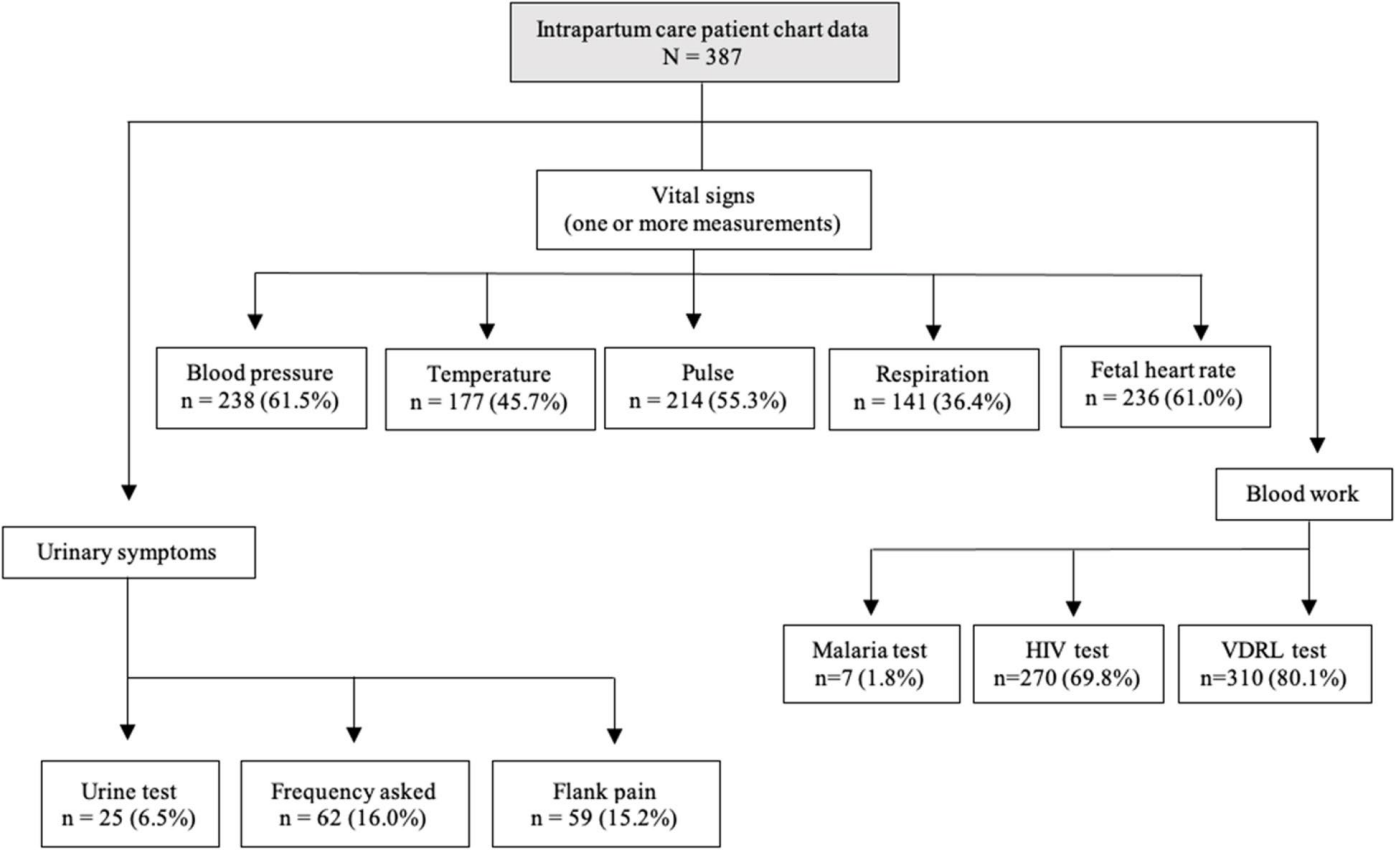
Patient chart diagnostics flow chart. Abbreviations: HIV (human immunodeficient virus), VDRL (venereal disease research laboratory test), IPTp (intermittent preventative treatemnts in pregnancy)

Figure 2 explores diagnostic data listed in the MR and IPR. Recorded vital signs ranged from 35.4% of women evaluated for respiratory rate to 61.5% of women evaluated for blood pressure. For blood work, 69.8% of women had a documented HIV test, 80.1% had a documented venereal disease research laboratory (VDRL) test, and 1.8% had a documented malaria test. For urinary symptoms, 6.5% of women had a documented urine test, 16.0% of women a documented urine frequency and/or pain while urinating, and 15.2% were documented as having been evaluated for flank pain.

Table 4 summarizes neonatal outcomes. Babies born with foetal distress had both the largest and the oldest babies with a mean birthweight of 2535.7 g and a mean GA of 34.7 weeks. Cases with antepartum stillbirth had both the smallest and the youngest babies with a mean birthweight of 1783.6 g and a mean GA of 31.5 weeks. For outcomes, foetal anaemia cases were the most likely to experience a transfer from the maternity ward to the NBU (OR 5.1, 95% CI 0.8, 30.9), whereas foetal distress cases were the least likely to transfer (OR 0.5, 95% CI 0.1, 2.5). Foetal anomaly had the highest odds of resulting in a PDM (OR 3.9, 95% CI 0.8, 19.2) whereas foetal distress and foetal anaemia had zero recorded PDMs.

**Table 4:** Neonatal outcomes of babies in preterm birth phenotyping categories

Phenotypes	n	BW (g)	GA (wks)	Parity	Age (yrs)	ANC (visits)	Referral (Newborn unit or other facility)			Pre-discharge mortality		
	**Means**						**%**	**OR**	**95 CI**	**%**	**OR**	**95 CI**
None	158	2343.4	34.4	1.3	22.0	3.3	22.0	0.9	[0.55, 1.44]	7.0	1.1	[0.5, 2.57]
Extrauterine infection	97	2223.9	33.8	1.9	24.6	3.3	24.7	1.1	[0.65, 1.91]	7.2	1.2	[0.48, 2.91]
Antepartum stillbirth	63	1783.6	31.5	2.1	24.3	2.2	-	-	-	-	-	-
Severe maternal condition	59	2348.4	34.1	1.7	24.8	3.3	20.7	0.8	[0.42, 1.66]	3.4	0.5	[0.11, 2.07]
Multiple gestation	47	2156.1	34.3	2.1	22.6	3.3	31.9	1.7	[0.85, 3.22]	8.5	1.4	[0.46, 4.31]
Preeclampsia	31	1876.3	32.5	1.7	24.2	3.2	22.6	1.0	[0.40, 2.31]	6.5	1.0	[0.22, 4.45]
Mid-to-late pregnancy bleeding	29	1872.1	32.2	2.3	26.0	3.8	37.9	2.2	[0.98, 4.76]	13.8	2.6	[0.82, 6.39]
Fetal distress	14	2535.7	34.7	2.4	25.6	3.5	14.3	0.5	[0.12, 2.46]	0.0	-	-
Fetal anomaly	10	1940.0	33.8	3.2	27.2	3.3	40.0	2.3	[0.62, 8.18]	20.0	3.9	[0.77, 19.17]
Chorioamnionitis	13	2126.9	32.1	3.5	27.5	2.5	30.8	1.5	[0.45, 4.95]	7.7	1.2	[0.15, 9.74]
Fetal anemia	2	2180.0	33.8	0.6	19.4	3.0	60.0	5.1	[0.84, 30.93]	0.0	-	-

*Abbreviations*: *BW* Birth weight, *GA* Gestational age, *ANC* Antenatal care

## Discussion

Adapting the Barros et al. preterm birth phenotyping framework allowed us to characterize preterm birth patterns at a county referral hospital. The use of routine data sources allowed for a novel retrospective analysis of an existing dataset, requiring less time and fewer resources than a prospective study. In our analysis of MCRH, we determined that extrauterine infection (most commonly HIV), antepartum stillbirth, and severe maternal conditions (most commonly anaemia) as the leading identifiable phenotypes of preterm birth. These findings are consistent with a recent systematic review of determinants of preterm birth in East Africa, can inform future prevention interventions in the region, and demonstrate the feasibility and utility of an adapted phenotyping framework applicable to lower-resource clinical settings in which the availability of clinical data is often limited [9].

The MCRH preterm rate of 11% is comparable to the burden across high-income and low-income settings with the recent Born Too Soon 2023 report showing a global preterm birth estimate of 9.9% [10]. With rates above 10% considered a high preterm birth burden, the 11% MCRH rate reflects the need for targeted prevention efforts.

The high percentage of unclassified preterm births (41%) was in line with the global trends and other phenotyping studies [3, 11]. In some settings this can reflect caregiver-initiated preterm labor due to less severe conditions or for iatrogenic reasons, however at MCRH this gap likely represents data entry gaps and errors or missed diagnoses. Plausible preterm birth diagnoses such as cervical or placental insufficiency were challenging to diagnose in this setting due to a lack of comprehensive obstetrical history and no availability of transvaginal ultrasound, dopplers, and other diagnostic tools.

Women in Migori County are at high risk for infection given HIV and malaria endemicity, lack of adequate sanitation in most households, and limited general healthcare accessibility [12]. Despite the WHO recommendation that all pregnant women in malaria endemic areas receive intermittent preventive treatment during pregnancy (IPTp), few charts included IPTp on patients’ medication lists, and few malaria tests were reported at the time of delivery [13]. This may be partially attributable to data documentation concerns, but still likely represents an area to target improvements. Low uptake of IPTp is consistent with other studies in East Africa and should be a high priority in the region [14, 15].

For women living with HIV in our dataset, viral load and antiretroviral therapy (ART) regimen information were unavailable, so it is impossible to know if active infection or the ART regimens themselves, as recent studies suggest, contributed to early onset of labor [16–21]. Women in Migori living with HIV and pregnant should receive careful prenatal monitoring, and oral preexposure prophylaxis (PrEP) should be considered for HIV negative women considered high risk for infection [13]. The relationship between ARTs and preterm birth also warrants further study.

Sexually transmitted and reproductive tract infections, including UTIs, are known to be highly correlated with preterm birth [22, 23]. Of women in our dataset, 6% had a UTI at the time of birth, and UTIs made up 25% of extrauterine infections. While UTI was not included in the Barros et. al phenotyping classification unless it had advanced to pyelonephritis, only 15% of patients in our cohort were evaluated for flank pain and only 6% received a urinalysis. We therefore included all UTIs to both account for potential missed diagnoses and to highlight the potential risk of UTIs even if they have not progressed to pyelonephritis [22, 24–28]. A recent study in Uganda concluded that the IPTp sulfadioxide was less effective in preventing malaria infection than an artemisinin-based regimen, but more effective at preventing preterm birth due to the broad-spectrum antimicrobial properties of sulfadioxine that likely treated persistent STIs and UTIs [29]. High rates of persistent UTIs have been document across various low-resource settings, implying the need for increased testing and treatment [25, 27].

The high incidence of maternal anaemia in our dataset may have multiple explanations. For one, anaemia is often associated with malaria or other parasitic infections and may indicate a higher incidence of malaria than reported [30–32]. For another, late pregnancy anaemia has been shown to be related to HIV seropositivity, including in a study conducted with pregnant women in western Kenya [33]. Finally, while maternal nutrition was not indicated in the charts, high rates of anaemia likely suggest a prevalence of iron deficiency and the need for nutritional counselling and assistance in the antenatal period [34, 35].

### Implications for clinical practice

The preterm birth phenotypes identified suggest the need for greater infection management in the antepartum and intrapartum periods for women in Migori County particularly malaria, UTIs, STIs, and HIV. Extrauterine infection was not only the largest identifiable phenotype but was the largest corresponding phenotype with both antepartum stillbirth and fetal distress. High rates of anaemia also suggest a need for increased routine monitoring and nutritional counselling and assistance.

These recommendations imply increased frequency and quality of ANC visits. In our MCRH dataset the mean number of ANC visits was only 3 compared to the WHO recommendation that all pregnant women receive at least 8 ANC visits [13]. It can be challenging for women to come to the clinic for ANC for myriad reasons, therefore options such as mobile clinics, in-home or community-based ANC, and incentive programs for women should be explored [13, 36–39]. Research has shown that distance from the health facility and an unwillingness to visit the facility alone are major factors limiting ANC visits [40].

### Implications for administrative practice

From a data perspective, enhanced linkage opportunities between maternity files and files from other departments (such as antenatal, newborn, and HIV wards) would give maternity healthcare workers critical information needed in the intrapartum period and could allow for a more comprehensive clinical picture to be documented in the chart. This would be most efficiently accomplished through the introduction of electronic medical record systems, but also through more consistent patient identification numerical systems in the interim.

### Limitations

Concessions were made in diagnostic precision to allow this model to be applied to births from facilities with limited infrastructure and data accuracy and completion concerns. As we were not diagnosing individuals for treatment but rather assigning phenotypes to look for facility trends our threshold for assigning a phenotype to a preterm birth was lowered. In a region in which necessary confirmatory tests are often missing and documentation in the medical record may be more limited, this approach allowed for greater inclusion of likely cases. With GA quality concerns, an adapted definition of preterm birth was used which may have under or over counted preterm babies (but was consistent with the definition used in the larger PTBi-EA study). Additionally, retrieving patient files for all eligible mothers was not possible due to poor filing systems. Only including births in which the IPR could be traced may have led to a level of selection bias, in that patients with complications may have been more likely to have proper documentation than those without. It is important to note, however, that the filing system at MCRH, is stronger than many others in the region; the high rates of IPRs that could be traced is a strength of this study. Finally, being unable to diagnose HELLP syndrome, IUGR, and early pregnancy bleeding is certainly a limitation. However, as the Barros et. al dataset reported no instances of HELLP syndrome and only a few cases of early pregnancy bleeding, we feel that the exclusion of IUGR was the biggest limitation in our analysis.

## Conclusions

Adapting the Barros et. al phenotyping framework allowed for an evaluation of preterm birth trends at MCRH that can inform future clinical and intervention strategies. This analysis highlighted the need for maternal extrauterine infection prevention and management in the antenatal and intrapartum periods for women in Migori. Even in regions where data is limited, our investigation demonstrates that adaptations can be made to the preterm phenotyping framework to accommodate a range in data accuracy and completeness. Indeed, areas with the highest preterm birth rates tend to also have the lowest availability of quality data, reinforcing the importance of adapting valuable methods to meet the needs of highly impacted communities.

## Data Availability

The datasets used and/or analysed for the current study are available from the corresponding author upon reasonable request.

## Abbreviations

ANC: Antenatal care
ART: Antiretroviral therapy
GA: Gestational age
GAPPS: Global Alliance to Prevent Prematurity and Stillbirth
IPR: Inpatient record
HELLP: Hemolysis, elevated liver enzymes and low platelets
HIV: Human immunodeficiency
IPTp: Intermittent preventive treatment during pregnancy
IUGR: Intrauterine growth restriction
KEMRI: Kenya Medical Research Institute
MCRH: Migori County Referral Hospital
MR: Maternity register
NBU: Newborn unit
OPK: Open Data Kit
PDM: Pre-discharge mortality
PPROM: Preterm premature rupture of membranes
PTBi-EA: East Africa Preterm Birth Initiative’s
RTI: Reproductive tract infection
UCSF: University of California, San Francisco
UTI: Urinary tract infection
VDRL: Venereal disease research laboratory
WHO: World Health Organization
